# A nationwide survey exploring physicians’ and pharmacists’ knowledge, awareness and perceptions regarding generic medicines in China

**DOI:** 10.1186/s12913-022-08438-9

**Published:** 2022-08-20

**Authors:** Jinghan Qu, Wei Zuo, Roxane L. Took, Kenneth W. Schafermeyer, Stephanie Lukas, Shaohong Wang, Liping Du, Xin Liu, Yang Gao, Jiantao Li, Hui Pan, Xiaoli Du, Dan Mei, Bo Zhang

**Affiliations:** 1 Department of Pharmacy, State Key Laboratory of Complex Severe and Rare Diseases, Peking Union Medical College Hospital, Chinese Academy of Medical Sciences and Peking Union Medical College Hospital, No.1 Shuaifuyuan Street, Beijing, Dongcheng District China; 2 Department of Pharmacy Practice, St. Louis College of Pharmacy, University of Health Sciences and Pharmacy in St. Louis, St. Louis, USA; 3 Department of Pharmaceutical and Administrative Sciences, St. Louis College of Pharmacy, University of Health Sciences and Pharmacy in St. Louis, St. Louis, USA; 4 Department of Medical Administration, State Key Laboratory of Complex Severe and Rare Diseases, Peking Union Medical College Hospital, Chinese Academy of Medical Sciences and Peking Union Medical College Hospital, Beijing, China

**Keywords:** Generic medicines, Generic substitution, Knowledge, Perceptions, Pharmacists, Physicians, Practices

## Abstract

**Background:**

Generic medicines substitution is an important means to control rapid growth of pharmaceutical expenditures for the healthcare system in China. Acceptance and utilization of generic medicines is highly influenced by healthcare providers’ perceptions. This study aimed to compare the knowledge, awareness and perceptions of generic medicines between physicians and pharmacists in China.

**Methods:**

We used an online, cross-sectional survey across China. The questionnaire explored four sections: demographic characteristics, assessment of the participants’ knowledge and awareness of generic medicines, perceptions of generic medicines and generic substitution practices. Chi-square or Mann–Whitney-U tests were applied to compare differences between physicians and pharmacists. *P*-values < 0.05 were considered significant.

**Results:**

A total of 1644 physicians and 4187 pharmacists participated. Most physicians (82.8%, *n* = 1362) and pharmacists (89.8%, *n* = 3760) correctly identified the definition of generic medicines. A similar percentage of physicians and pharmacists agreed that approved generic medicines are as effective (64.1% vs 68.2%) or safe (63.8% vs 69.1%) as brand-name medicines. Most physicians and pharmacists (67.6% vs 71.0%) supported the policy of generic substitution. In practice, 79.4% (*n* = 1305) of physicians reported that they had prescribed generic medicines. More than 78% of respondents reported an obvious increase in the number of generic medicines prescribed in their medical institutions. The majority of physicians and pharmacists identified lack of trust regarding efficacy and safety of generic medicines and the difficulty of changing patients’ preference as top challenges in generic substitution.

**Conclusions:**

Both physicians and pharmacists surveyed had adequate knowledge of generic medicines, and hold positive attitude towards generics and generic substitution. Efficacy and safety are key factors related to prescribing or dispensing generic medicines. Various policies and regulations should be taken to encourage successful generic substitution.

**Supplementary Information:**

The online version contains supplementary material available at 10.1186/s12913-022-08438-9.

## Background

Total pharmaceutical expenditures in China continue to increase at a faster growth rate each year [1–3]. Generic substitution is one important means for controlling rapid growth of pharmaceutical expenditures for healthcare systems. The National Medical Products Administration (NMPA) of China defines generic medicine products as those have the same active ingredients, dosage forms, routes of administration and therapeutic effects as their brand equivalents [4, 5]. In March 2016, NMPA stipulated that generic medicines approved before 2007 are subject to consistency evaluation through bioequivalence trials [4]. The standard criteria of bioequivalence stipulate that two treatments are not different from on another if two pharmacokinetic parameters – peak concentration (Cmax) and the area under concentration–time curve (AUC) – fall entirely within 90% confidence interval (*i.e.*, the range of 80.00%-125.00% of the mean) [6].

In January 2019, the General Office of the State Council of China implemented the national centralized drug procurement program in 11 selected locations, in order to improve medication accessibility, reduce medicine prices and contain the sharp increase in healthcare expenses. The procurement program was based on volume purchasing. As part of this process, drug enterprises bid or negotiate for the specific quantity and finally determine the purchase price [7–10]. The selected generic medicines need to pass the NMPA evaluation for the consistency of quality and efficacy [11].The pharmaceutical companies won the contract in the bid to provide the medication for one or three years. In order to expand the effects of the volume-based purchasing pilot program, the state organized the next four batches of national centralized procurement in the year 2020 and 2021. A total of 218 drugs involving about 220 billion yuan were selected, most of which were for treatment of chronic diseases. The prices on average were reduced by 56%, with an estimated 92 billion yuan saving [12–17]. With expansion of the national centralized procurement program, this has become the dominant mode of drug purchasing in China.

Around the globe, generic substitution policy has been adopted in many healthcare systems [18–22]. According to the report from the Food and Drug Administration (FDA), almost 9 out of 10 prescriptions filled in the United States are for generic medicines [23]. The prices of generic medicines are almost 85% less than the brand-name due to market competition between multiple generic companies. Generic medicines saved the U.S. health care system $2.2 trillion from 2009 to 2019 [24]. In Europe, generic medicines constitute nearly 67% prescriptions dispensed but only account for only 29% of the total expenditure on medicines. It was estimated that the use of generic medicines reduces the overall cost of the health care in Europe by €100 billion annually [25]. The share of generic medicines in the market differs between countries, some countries like Spain, Greece and Brazil hold small share [26–29]. The reasons for the low use of generic medicines are negative perception about the quality of them, lack of consumer knowledge, hurdles of drug identification and so on [30–34]. A number of measures are established to promote use of generic medicines, including demand-side policies, educational efforts in patients’ and prescribers’ confidence and knowledge about these medicines, close surveillance for good manufacturing practices, quality control for medication [35–39]. Howard et. al. conducted a review about influencers of generic medicines utilization, presenting that physicians and pharmacists play an important role in generic medicines use [40]. Guttier et. al. conducted a systematic review on the impact of interventions to promote the use of generic medicines, showing that educational, financial incentives and other interventions applied to prescribers and pharmacists had impact on the increase use of generics [41].

There have been a number of studies in various locations assessed opinions, knowledge, attitudes and awareness of generic medicines amongst healthcare professionals [31, 42–49]. However, there were few literatures reporting this in China. Currently, with the implementation of the procurement program in China, all localities and departments have increased use of the selected medicines, however, there are still concerns among healthcare providers regarding drug quality. Acceptance and utilization of generic medicines is highly influenced by healthcare providers’ perceptions. Thus, the primary objective of this research was to investigate and compare the knowledge, awareness and perceptions of physicians and pharmacists regarding generic medicines in China.

## Methods

### Study design

An online cross-sectional survey was conducted on China’s mainland between April and May 2020. Physicians and pharmacists working in hospitals or communities were included in this study.

### Survey instrument

The first draft of the questionnaire was formed with Chinese language after extensive literature search and review, peer-reviewed and examined by a panel of experts for content and construct validity test. The panel of experts contained 3 physicians, 3 pharmacists, 2 statisticians, 2 experts in hospital administration. Based on the feedback obtained from experts, three items- the type of medical institutions, the secondary department and the knowledge of policy on generic medicines were deleted, and ambiguities were resolved. Then the questionnaire was pilot tested among 30 physicians and pharmacists in Beijing. The data of the pilot study (see Additional file 1. Table S1 and 2) were not included in the final analysis. Minor textual changes were made to form the final version of the survey instrument.

The final questionnaire (see Additional file 2) was created on the Wenjuanxing website and comprised four sections: general characteristics of the participants, assessment of the participants’ knowledge and awareness, perceptions of participants towards generic medicines and generic substitution practices. For physicians, the second section consisted of four knowledge/awareness-based questions regarding the national procurement program and generic medicines. For pharmacists, this section included an additional question asking whether participants were aware of the phrase “This product has passed the consistency evaluation” on the generic products. In this second section of the questionnaire, a “yes” or correct response was scored 1 point, whilst a “no”, incorrect or unclear response was scored 0 point. The maximum score on the knowledge and awareness section was 4 for physicians and 5 for pharmacists. The third section on perceptions of generic medicines consisted of 10 statements using a five-point Likert type scale: 1 = strongly disagree, 2 = disagree, 3 = neutral, 4 = agree, 5 = strongly agree. For statistical reasons, the fifth statement was reverse scored from 1 = strongly agree to 5 = strongly disagree. The total perception score is out of 50, with 50 being strongly agree with all questions. The fourth section addressed the perceptions of generic substitution practices. This included 7 items for physicians or 5 items for pharmacists.

### Data collection

The questionnaires were distributed via the WeChat messaging app across China through an open-access hyperlink. The questionnaire was available for two weeks. Data were automatically collected via Wenjuanxing when the participant completed the questionnaire. Only completed questionnaires were included.

### Inclusion and exclusion criteria

Physicians and pharmacists in both hospitals and primary care settings were recruited. Data from other health care providers were excluded based on the Question-1related to the occupation in the questionnaire. Participation in the survey was voluntary; no incentive was provided for enrollment of participants.

### Statistical analysis

The data collected were analyzed using SPSS version 24. Frequency, percentage and median were calculated to express different variables. Kolmogorov–Smirnov test was used to determine normality of the data. Chi-square test or Mann–Whitney-U was applied to measure the association between physicians and pharmacists. *P*-values < 0.05 were considered significant. Reliability analysis (Cronbach alpha coefficient) of items focused on the perceptions towards generic medicines was applied.

## Results

A total of 6164 health care providers (1644 physicians, 4187 pharmacists and 333 other professionals) completed the questionnaire. A total of 5831 questionnaires from physicians and pharmacists were included in the analysis, data from other professionals were excluded. Demographic details are presented in Table 1.

**Table 1:** Demographic characteristics of participated physicians and pharmacists

Characteristics	Frequency (%)	
	**Physicians *n* = 1644**	**Pharmacists *n* = 4187**
Age(y)		
20–29	140 (8.5)	694 (16.6)
30–39	425 (25.9)	1944 (46.4)
40–49	614 (37.3)	1030 (24.6)
50–59	434 (26.4)	505 (12.1)
≥ 60	31 (1.9)	14 (0.3)
Gender		
Male	744 (45.3)	1236 (29.5)
Female	900 (54.7)	2951 (70.5)
Terminal degree		
PhD	413 (25.1)	124 (3.0)
Master	404 (24.6)	747 (17.8)
Bachelor	727 (44.2)	2768 (66.1)
Others	100 (6.1)	548 (13.1)
Professional title		
Professor of medicine/pharmacy	490 (29.8)	290 (6.9)
Associate professor of medicine/pharmacy	449 (27.3)	663 (15.8)
Doctor/Pharmacist in charge	438 (26.6)	1620 (38.7)
Doctor/Pharmacist	223 (13.6)	1518 (36.3)
No title (e.g. Intern)	36 (2.2)	89 (2.1)
others	8 (0.5)	7 (0.2)
Years of experience		
Less than 5	168 (10.2)	754 (18.0)
6–10	238 (14.5)	1146 (27.4)
11–20	423 (25.7)	1013 (24.2)
21–30	526 (32.0)	900 (21.5)
More than 30	289 (17.6)	374 (8.9)
Level of medical institution		
Tertiary hospital	1113 (67.7)	3267 (78.0)
Secondary hospital	243 (14.8)	708 (16.9)
Community hospital	72 (4.4)	46 (1.1)
Primary health care institution	216 (13.1)	166 (4.0)

### Knowledge and awareness of generic medicines

The mean knowledge and awareness score of physicians was 2.50 (SD = 0.87) out of the total obtainable score of 4, compared to 2.80 (SD = 0.74) out of the total score of 5 among pharmacists. There were statistically significant differences between the physicians and pharmacists in terms of their responses to each knowledge statement (*P* < 0.001) as shown in Table 2.

**Table 2:** Knowledge and awareness of generic medicines

Statement	Yes or Correct response n (%)		No or Incorrect response n (%)		Unsure n (%)		*P*-value ^*^
	**Physicians**	**Pharmacists**	**Physicians**	**Pharmacists**	**Physicians**	**Pharmacists**	
Were you aware that China carries out the program of quality and efficacy consistency evaluation of generic medicines?	1349 (82.1)	3828 (91.4)	161 (9.8)	159 (3.8)	134 (8.2)	200 (4.8)	< 0.001
Were you aware of the logo “Have passed the Consistency Evaluation” on the generic products?	N/A	3039 (72.6)	N/A	630 (15.0)	N/A	518 (12.4)	N/A
True/False: In principle, the method of bioequivalence tests in vivo is used for Consistency Evaluation. The standard of bioequivalence is that the 90% confidence interval of the geometric mean experiment/ reference ratios for main pharmacokinetic parameters (Cmax and AUC) falls entirely within the range of 90.00% ~ 120.00%	41 (2.5)	405 (9.7)	1289 (78.4)	2996 (71.6)	314 (19.1)	786 (18.8)	< 0.001
Were you aware that all the generic medicines in national centralized procurement have passed the consistency evaluation of quality and efficacy?	1366 (83.1)	3710 (88.6)	63 (3.8)	156 (3.7)	215 (13.1)	321 (7.7)	< 0.001
True/False: The generic medicines in the national centralized procurement have the same active ingredients, dosage forms, routes of administration and therapeutic effects with the brand-name medicines	1362 (82.8)	3760 (89.8)	70 (4.3)	134 (3.2)	212 (12.9)	293 (7.0)	< 0.001

*N/A* Not applicable

^*^P-value calculated using chi-square test

The majority of physicians (82.1%, *n* = 1349) were aware that China carries out the program of quality and efficacy consistency evaluation of generic medicines; by contrast, a higher percentage (91.4%, *n* = 3828) of pharmacists reported they were aware. Additionally, 72.6% (*n* = 3039) of pharmacists stated that they were familiar with the consistency evaluation logo printed on generic products. Only 2.5% (*n* = 41) of physicians and 9.7% (*n* = 405) of pharmacists correctly identified standard criteria of bioequivalence in the consistency evaluation.

The majority of physicians (83.1%, *n* = 1366) and pharmacists (88.6%, *n* = 3710) stated that they were aware that all the generic medicines in the national centralized procurement had passed the consistency evaluation. Similarly, most physicians (82.8%, *n* = 1362) and pharmacists (89.8%, *n* = 3760) knew the definition of generic medicines.

### Perceptions regarding generic medicines

The Cronbach’s alpha value for perceptions is equal to 0.748. The total perceptions score (*P* < 0.05) was proven non-normally distributed using Kolmogorov–Smirnov test. The mean of total perception score was 36.68 (SD = 5.08) for physicians, 36.99 (SD = 4.46) for pharmacists (*P* = 0.204), indicating no statistically significant difference between pharmacists and physicians. The results showed significant difference in the tertiary hospitals, the mean of total perception score was 36.46 (SD = 5.00) for physicians, 37.00 (SD = 4.46) for pharmacists (*P* < 0.001). Comparisons of perceptions regarding generic medicines among different demographic characteristics were shown in the Additional file 3 Table S3.

**Fig. 1:**
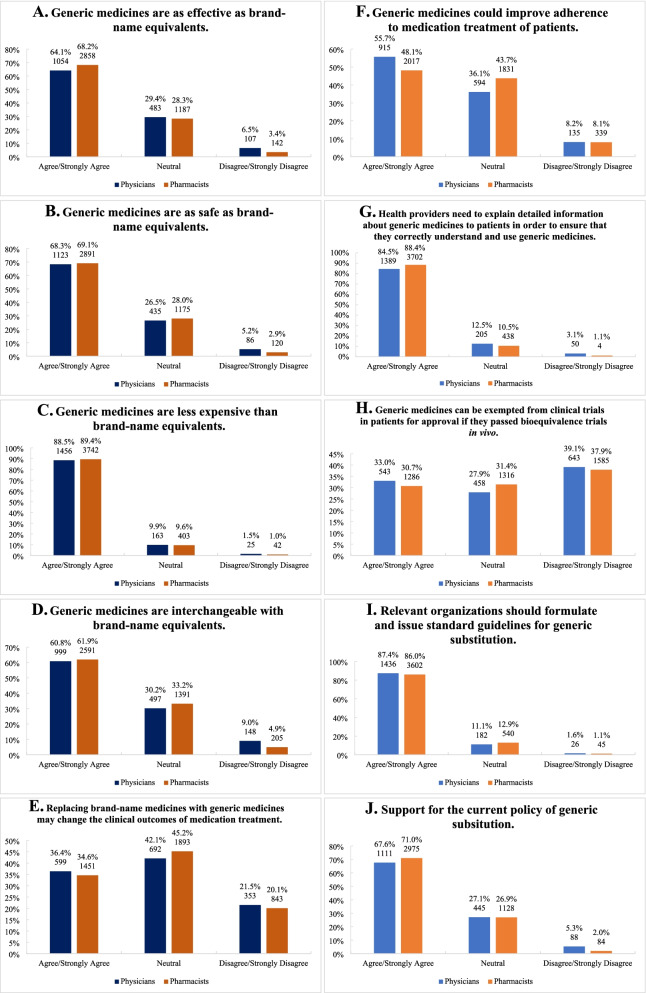
Perceptions toward generic medicines

Large numbers of physicians (64.1%, *n* = 1054) and pharmacists (68.2%, *n* = 2858) agreed or strongly agreed generic medicines that have passed the consistency evaluation are as effective as brand-name equivalents (Fig. 1A). A similar percentage of physicians and pharmacists (68.3%, *n* = 1123 and 69.1%, *n* = 2891, respectively) agreed that generic medicines are as safe as brand-name medicines (Fig. 1B). Also, similar results showed that physicians and pharmacists (88.5%, *n* = 1456 and 89.4%, *n* = 3742, respectively) believed generic medicines are cheaper than innovators (Fig. 1C). Approximately 61% physicians and pharmacists agreed that generic medicines are interchangeable with brand-name equivalents (Fig. 1D).

Nearly half of physicians and pharmacists (42.1%, *n* = 692 and 45.2%, *n* = 1893, respectively) hold neutral perceptions about replacing brand-name medicines with generics, citing concerns regarding varied clinical outcomes of medication treatment (Fig. 1E). Seven percent more physicians versus pharmacists believed generic substitution improve adherence to medication treatment (Fig. 1F).

The majority of physicians and pharmacists (84.5%, *n* = 1389 and 88.4%, *n* = 3702, respectively) expressed a need for explaining detailed information about generic medicines to patients when considering generic substitution (Fig. 1G). Several physicians and pharmacists (39.1%, *n* = 643 and 37.9%, *n* = 1585, respectively) felt that clinical trials in patients still should not exempt, although generics passed bioequivalence trials in vivo (Fig. 1H).

Overall, most physicians and pharmacists (67.6%, *n* = 1111 and 71.0%, *n* = 2975, respectively) support policy of generic substitution (Fig. 1J). Perception of physicians and pharmacists regarding generic medicines can be found in Fig. 1A-J.

### Perceptions of generic substitution practices

In practice, 79.4% (*n* = 1305) physicians reported that they prescribed generic medicines at some point in their career. For the newly diagnosed patients, 73.1% of the physicians were favorable in prescribing generic medicines as a priority, and those physicians advised 64.79% (SD = 24.66) of the newly diagnosed patients to use generic medicines, while about 60.96% (SD = 23.48) of these patients accepted their recommendation regarding generic substitution. For established patients that have already received medication treatment, 63.5% of physicians were in favor of prescribing generic medicines as a priority, and those physicians advised 60.89% (SD = 25.63) of the established patients to take generic medicines, while 54.92% (SD = 23.99) of these patients accepted the physicians’ recommendation.

In addition, 79.9% (*n* = 1314) physicians reported a significant decrease in the amount of brand-name medicines prescribed and an increase in the amount of generic medicines used in their medical institutions; only 4.9% (*n* = 81) stated otherwise. When pharmacists were surveyed about generic medicine use in their medical institution since the implementation of national centralized procurement, 78.0% (*n* = 3264) noted an increase in generics use.

**Fig. 2:**
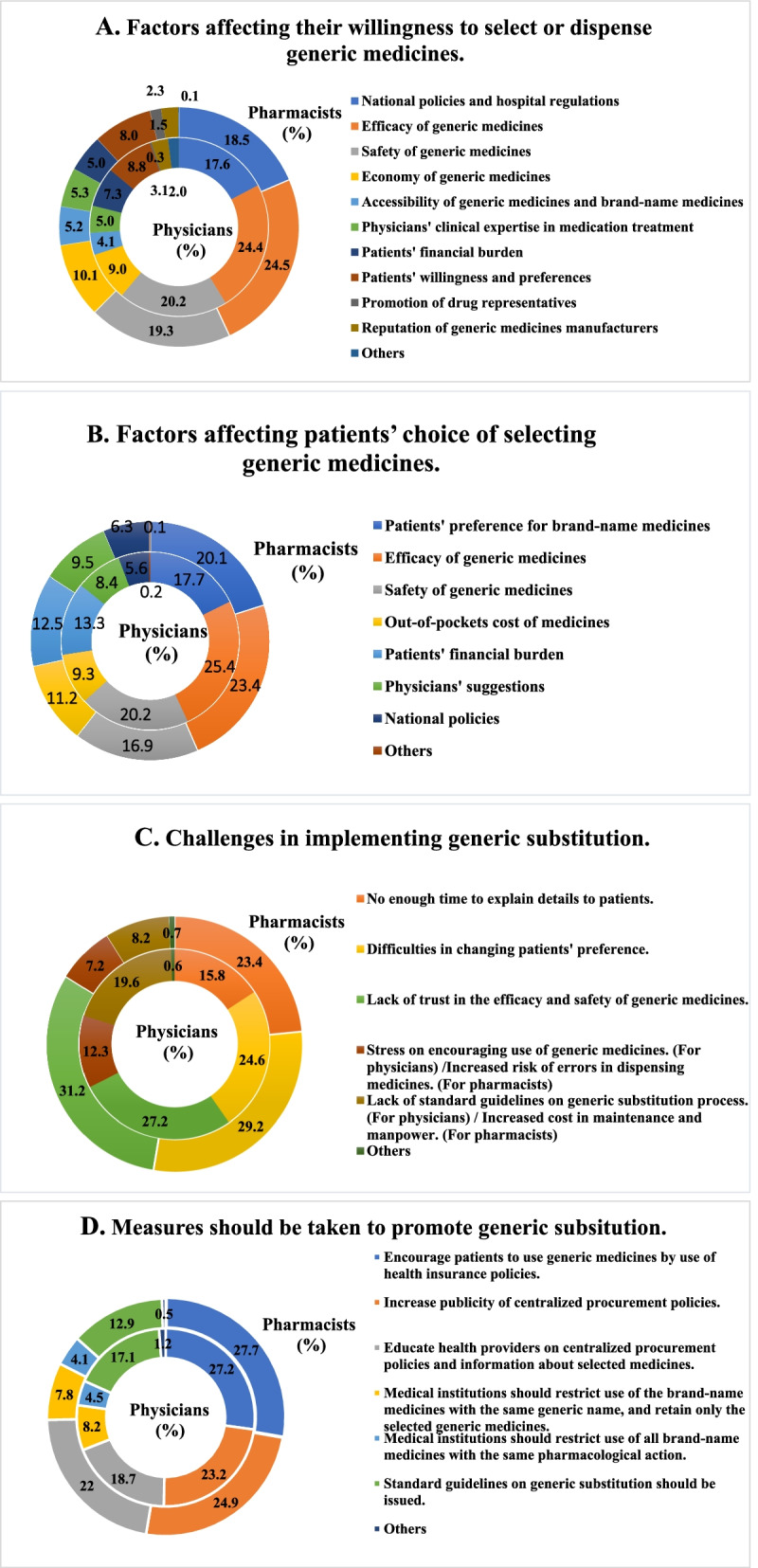
Perceptions of generic substitution practices

The majority of the physicians and pharmacists indicated that efficacy and safety of the medication, as well as national policies and hospital regulations were the most important considerations when prescribing or dispensing generics. Only 7.3% physicians and 5.0% pharmacists regarded patients’ financial burden as an important factor in generics use. It is interesting to note that 0.3% (*n* = 15) of physicians, comparing 1.5% (*n* = 194) of pharmacists believed drug representatives played a vital role in generic medicines use (Fig. 2A).

Physicians and pharmacists believed that efficacy and safety of the generic medicines, and preference for brand-name medicines were the most important factors for patients consenting to generic medicines use. Physicians were more likely to prioritize generic medicines safety versus patients’ preference for brand-name medicines (Fig. 2B).

The majority of physicians and pharmacists considered lack of trust in the efficacy and safety of generic medicines and difficulty of changing patients’ preference as top challenges in implementing generic substitution. Moreover, several physicians articulated lack of standard guidelines on generic substitution process (19.6%, *n* = 966), lack of time to explain details to patients (15.8%, *n* = 778), and stress on generic medicines initiative (12.3%, *n* = 606) as other challenges to implementation of the procurement program. Pharmacists indicated that a lack of time to explain to generic substitution to patients (23.4%, *n* = 2945), increased cost for manpower (8.2%, *n* = 1035) and higher risk of dispensing errors (7.2%, *n* = 901) as other challenges (Fig. 2C).

The majority of physicians and pharmacists indicated that generic substitution could be promoted by generic preference by health insurance policies, increasing publicity, and educating health providers about these generic selection policies. A larger percentage of physicians (17.1%, *n* = 842), expressed need for standard guidelines on generic substitution versus pharmacists (12.9%, *n* = 1619) (Fig. 2D).

## Discussion

This survey is a nationwide survey of physicians and pharmacists regarding generic medicines in the national centralized procurement evaluating the knowledge and awareness, perceptions of generic medicines and generic substitution practices. The Cronbach’s alpha value for perceptions is equal to 0.748, indicating a good level of reliability.

According to the results, both physicians and pharmacists generally have good knowledge and awareness of generic medicines, great degree of positive perceptions and acceptance of generic substitution. Similar findings were reported in several systematic reviews [30, 31, 45]. Toverud et al. found that pharmacists and physicians determine the quality of generic medicines based on the maturity of the healthcare system. Authors concluded that mature healthcare systems have more reliable public control routines for medicines in general as well as better bioequivalence requirements concerning generics, which promotes support for generic medicines use [44].

In terms of efficacy and safety, nearly one third of our survey respondents maintained a neutral stance. This could be attributed to the fact that the national procurement program had only been implemented for approximately 18 months prior to the survey. Healthcare providers might feel there is still a lack of research data to prove the efficacy and safety in clinical settings, especially for chronic diseases. Another reason for the uncertainty regarding efficacy and safety may be that variability among patients and/or generics could hinder confidence of healthcare providers. Local manufacturers of generic medicines might have not yet gained full trust of the public. As some literature has showed, physicians are highly concerned about the manufacturers’ trustworthiness [50–52]. Kuribayashi et al. clarified modernization and strengthening of bioequivalence guidelines in Japan, and compared those requirements with that of the Pharmaceuticals and Medical Devices Agency (PMDA), the European Medicines Agency (EMA) and the FDA [53]. We recommend the Chinese government make stringent standards for bioequivalence concerning generic medicines, supervise quality control for generics, take regulatory efficacy and safety action at the post-market stage, and create fair competition mechanisms to promote reasonable prices and high quality [54–57]. Physicians may prefer to prescribe brand-name medicines because they are more familiar with these products [58]. Official data about generics and their quality should be recorded and published to build public trust regarding generics use.

Physicians have a key role in prescribing generic medicines. Pharmacists are on the front line dispensing medicines according to medical prescription and are the last medical professionals that impact medication selection [30, 59]. It is crucial to establish collaboration between physicians and pharmacists in order to improve generic substitution [60, 61]. The respondents of our study advocated the necessity to collaboratively educate patients about generic medicines to help eliminate misconceptions.

The availability and use of fairly priced generic medicines make an important contribution in improving generic substitution [62, 63]. In China there is no price regulation or reimbursement policy for promoting generic medicine use. In small cities, drug expenditures in the outpatient setting are not covered by health insurance. Patients prefer to purchase the more affordable product, however, in large cities, there is less difference in reimbursement between brand products and generics. Patients just need to pay a small out-of-pocket expense, so they are more likely to choose brand-name medicines. Taking this into consideration, policymakers promoting generic substitution should take into account local conditions.

In our study, it was noted that approximately half of the physicians and pharmacists believed generic medicines affect patients’ adherence to therapeutic regimens. With increasing population age, the prevalence of chronic diseases has also increased in China. Up to 75.8% of residents ≥ 60 years of age have at least one chronic disease [64]. Patients with chronic diseases need to take medicines long-term, often for the duration of their life, so affordable medicines decrease healthcare spending and encourage patients to adhere to medication therapy [29, 65, 66].

It was interesting to find that most physicians consider generic medicines more suitable for newly diagnosed patients, and that physicians felt newly diagnosed patients would be more likely to accept a prescription for a generic medicine. Patients tend to use the same medicine during their course of treatment, if a new product differs in manufacturer, shape or color, patients easily become confused, especially for elderly patients and polypharmacy users [30, 67–69]. This may explain why physicians find it difficult to challenge preconceived notions to persuade patients with prior brand-name use. Therefore, it is vital for manufacturers to ensure adequate, consistent supply of generic medicines on the national centralized procurement list, so that physicians and pharmacists could minimize changes for patients.

There is an urgent need for a clear, standard generic substitution process in clinical settings. Some respondents argued that there is mismatch between medicines recommended by the formal clinical guidelines with those listed in the national centralized procurement program. Therefore, professional organizations should make recommendations to Chinese health authorities regarding a standard protocol for generic medicines use. All in all, various policies and regulations relation to prescribing, dispensing, patients/consumers, and healthcare organizations could be taken into consideration to encourage successful generic subsitution [70]. These findings have important implications for the promotion of generic substitution.

## Limitation of the study

This study was a descriptive survey using a convenience widely-spreading sampling technique, with which we cannot ensure samples randomly selected from the subject pool, nor compute the total number of the target population or a response rate. The volume of responses varied among regions of China. These limitations may weaken the results on the statistical significance of the differences and correlations presented about the data. Moreover, the use of a self-administered questionnaire, relied on the honesty, faith and patience of the participants and may have been subject to response or recall bias.

## Conclusions

This study shows that both physicians and pharmacists have a fairly adequate knowledge and awareness of generic medicines, and tend to hold positive perceptions and acceptance of generics and generic substitution. Efficacy and safety are the key factors related to prescribing or dispending generic medicines. Changes in health insurance policies, increasing publicity and educating health providers may promote usage and acceptance of generic medicines.

## Supplementary Information

**Additional file 1:** **Table S1.** Demographic characteristics of participated physicians and pharmacists in the pilot study. Table S2. Data of the pilot study.

**Additional file 2:** Knowledge, Awareness and Perceptions of Healthcare Providers Regarding Generic Medicines in China.

**Additional file 3:** **Table S3.** Perceptions regarding generic medicines between physicians and pharmacists.

## Data Availability

The datasets used and/or analyzed during the current study are available from the corresponding author on reasonable request.

## Abbreviations

NMPA: National Medical Products Administration
Cmax: Peak concentration
AUC: The area under concentration–time curve
FDA: Food and Drug Administration
PMDA: Pharmaceuticals and Medical Devices Agency
EMA: European Medicines Agency
