# Supplementary material for: A nationwide survey exploring physicians’ and pharmacists’ knowledge, awareness and perceptions regarding generic medicines in China
Source: BMC Health Serv Res. 2022 Aug 20;22:1069. doi: 10.1186/s12913-022-08438-9 (PMC9392939; doi:10.1186/s12913-022-08438-9)
Supplement: Supplementary file 1 — Additional file 1: Table S1. Demographic characteristics of participated physicians and pharmacists in the pilot study. Table S2. Data of the pilot study. [file 12913_2022_8438_MOESM1_ESM.docx]

**Table S1. Demographic characteristics of participated physicians and pharmacists in the pilot study.**

| **Characteristics** | **Frequency (%)** | |
| --- | --- | --- |
|  | **Physicians**  **n=16** | **Pharmacists**  **n=14** |
| Age(y) | | |
| 20-29 | 3 | 2 |
| 30-39 | 4 | 4 |
| 40-49 | 4 | 4 |
| 50-59 | 3 | 2 |
| ≥60 | 2 | 2 |
| Gender | | |
| Male | 7 | 4 |
| Female | 9 | 10 |
| Terminal degree | | |
| PhD | 7 | 3 |
| Master | 5 | 6 |
| Bachelor | 3 | 4 |
| Others | 1 | 1 |
| Professional title | | |
| Professor of medicine/pharmacy | 5 | 3 |
| Associate professor of medicine/pharmacy | 5 | 4 |
| Doctor/Pharmacist in charge | 3 | 4 |
| Doctor/Pharmacist | 2 | 2 |
| No title (e.g. Intern) | 1 | 1 |
| others | 0 | 0 |
| Years of experience | | |
| Less than 5 | 3 | 3 |
| 6-10 | 3 | 4 |
| 11-20 | 5 | 3 |
| 21-30 | 3 | 2 |
| More than 30 | 2 | 2 |

**Table S2. Data of the pilot study.**

|  | **Physicians**  **n=16** | **Pharmacists**  **n=14** |
| --- | --- | --- |
| Total score of knowledge (Mean ± SD) | 2.63 ± 0.857 | 3.93 ± 0.593 |
| Total score of perceptions (Mean ± SD) | 23.44 ± 4.330 | 21.64 ± 5.121 |
| Cronbach’s alpha value for perceptions | 0.721 | 0.867 |
|  | 0.818 | |
