# Supplementary material for: A nationwide survey exploring physicians’ and pharmacists’ knowledge, awareness and perceptions regarding generic medicines in China
Source: BMC Health Serv Res. 2022 Aug 20;22:1069. doi: 10.1186/s12913-022-08438-9 (PMC9392939; doi:10.1186/s12913-022-08438-9)
Supplement: Supplementary file 2 — Additional file 2: Knowledge, Awareness and Perceptions of Healthcare Providers Regarding Generic Medicines in China. [file 12913_2022_8438_MOESM2_ESM.docx]

**Knowledge, Awareness and Perceptions of Healthcare Providers Regarding Generic Medicines in China**

**Part I: Demographic characteristics.**

1. What is your occupation?

A. Physician

B. Pharmacist

C. Other: ____________

2. Which of the following range does your age fall in?

A. 20-29 years old

B. 30-39 years old

C. 40-49 years old

D. 50-59 years old

E. Over 60 years old

3. What is your gender?

A. Male

B. Female

4. What is your terminal education degree?

A. PhD

B. Master degree

C. Bachelor degree

D. Others

5. What is your professional title?

A. Professor of medicine/pharmacy

B. Associate professor of medicine/pharmacy

C. Doctor/Pharmacist in charge

D. Doctor/Pharmacist

E. No title (e.g. Intern)

F. Others

6. By the end of March 2020, how many years have you worked as a physician or a pharmacist?

A. Less than 5 years

B. 6-10 years

C. 11-20 years

D. 21-30 years

E. Over 30 years

7. Where are you from?

City __________, P[rovince](file:///D:\Program%20Files%20(x86)\Dict\Dict\7.5.2.0\resultui\dict\?keyword=province)__________

8. What is the level of your medical institution?

A. Tertiary hospital

B. Secondary hospital

C. Community hospital

D. Primary healthcare institutions (including community health service center, township health center, village health office, clinics)

**Part II: Knowledge and awareness of generic medicines.**

9. Were you aware that China carries out the program of quality and efficacy consistency evaluation of generic medicines? (hereinafter referred to as "Consistency Evaluation")?

A. Yes

B. No

C. Unsure

10. Were you aware of the logo "Have passed the Consistency Evaluation" on the generic products? (Only for pharmacists)

A. Yes

B. No

C. Unsure

11. For the standard criteria of bioequivalence, please judge whether the following statements are correct or not.

*In principle, the method of bioequivalence tests in vivo is used for Consistency Evaluation. The standard of bioequivalence is that the 90% confidence interval of the geometric mean experiment/ reference ratios for main pharmacokinetic parameters (Cmax and AUC) falls entirely within the range of 90.00% ~ 120.00%.*

A. True

B. False

C. Unsure

12. Were you aware that all the generic medicines in national centralized procurement have passed the consistency evaluation of quality and efficacy?

A. Yes

B. No

C. Unsure

13. Please judge whether the following statement is correct or not.

*The generic medicines in the national centralized procurement have the same active ingredients, dosage forms, routes of administration and therapeutic effects with the brand medicines.*

A. True

B. False

C. Unsure

**Part III: Perceptions toward generic medicines.**

14. Generic medicines that have passed the consistency evaluation are as effective as brand-name equivalents.

□Strongly agree □Agree □Neutral □Disagree □Strongly disagree

15. Generic medicines that have passed the consistency evaluation are as safe as brand-name equivalents.

□Strongly agree □Agree □Neutral □Disagree □Strongly disagree

16. Generic medicines that have passed the consistency evaluation are less expensive than brand-name equivalents.

□Strongly agree □Agree □Neutral □Disagree □Strongly disagree

17. Generic medicines that have passed the consistency evaluation are interchangeable with brand-name medicines.

□Strongly agree □Agree □Neutral □Disagree □Strongly disagree

18. Replacing brand-name medicines with generic medicines that passed the consistency evaluation may change the clinical outcomes of medication treatment.

□Strongly agree □Agree □Neutral □Disagree □Strongly disagree

19. Application of generic medicines that passed the consistency evaluation could improve adherence to medication treatment of patients.

□Strongly agree □Agree □Neutral □Disagree □Strongly disagree

20. Health providers need to explain detailed information about generic medicines to patients in order to ensure that they correctly understand and use generic medicines.

□Strongly agree □Agree □Neutral □Disagree □Strongly disagree

21. Generic medicines can be exempted from clinical trials for approval if they passed bioequivalence trials in vivo.

□Strongly agree □Agree □Neutral □Disagree □Strongly disagree

22. Relevant organizations should formulate and issue standard guidelines for generic substitution.

□Strongly agree □Agree □Neutral □Disagree □Strongly disagree

23. I support the current policy of substituting brand-name medicines with generic medicines that have passed the consistency evaluation.

□Strongly agree □Agree □Neutral □Disagree □Strongly disagree

**Part IV: Perceptions of generic substitution practices.**

24. Only for physicians.

1). For the newly diagnosed patients, would you prefer to prescribe generic medicines as priority?

A. Yes.

B. No. (Jump to Question 25 directly)

2). What’s the proportion of the newly diagnosed patients you advise to use generic medicines? _______

3). What’s the proportion of the patients accepted your advice to use generic medicines? _______

25. Only for physicians.

1). For the established patients that have already received medication treatment, would you prefer to prescribe generic medicines as priority?

A. Yes.

B. No. (Jump to Question 26 directly)

2). What’s the proportion of the patients that have already received medication treatment you advise to use generic medicines? _______

3). What’s the proportion of the patients accepted your advice to use generic medicines? _______

26. How has the amount of generic medicines used in your medical institution changed after the implementation of national centralized procurement of medicines?

A. Significantly increased

B. Increased somewhat

C. Basically unchanged

D. Decreased

E. Unsure

27. What factors do you think affect your selection of generic medicines? Please select the top 3 important items.

□ National policies and hospital regulations

□ Efficacy of generic medicines

□ Safety of generic medicines

□ Economy of generic medicines

□ Accessibility of generic medicines and brand-name medicines

□ Physicians' clinical expertise in medication treatment

□ Patients' financial burden

□ Patients' willingness and preferences

□ Promotion of drug representatives

□ Reputation of generic medicines manufacturers

□ Other:__________

28. What factors do you think affect patients’ choice of selecting generic medicines in the national centralized procurement? Please select the top 3 important items.

□ Patients' preference for brand-name medicines and medication habits

□ Efficacy of generic medicines

□ Safety of generic medicines

□ Out-of-pockets cost of medicines

□ Patients' financial burden

□ Physicians' suggestions

□ National policies

□ Other:__________

29. What do you think is the largest challenge in implementing the national centralized procurement and use of generic medicines? Please select the top 3 important items.

□ There is no enough time to explain details to patients.

□ It is difficult to change patients' preference.

□ Lack of trust in the efficacy and safety of generic medicines.

□ There is an increased risk of errors in dispensing medicines.

□ There is an increased cost in maintenance and manpower.

□ Other:__________

­­30. What measures should be taken to promote the national centralized procurement and use of generic medicines? Please select the top 3 important items.

□ Encourage patients to use generic medicines by use of health insurance policies.

□ Increase publicity of centralized procurement policies.

□ Educate health providers on centralized procurement policies and information about selected medicines.

□ Medical institutions should restrict the use of the brand-name medicines with the same generic name, and retain only the selected generic medicines.

□ Medical institutions should restrict the use of all brand-name medicines with the same pharmacological action.

□ Standard guidelines on generic substitution should be issued.

□ Other:_________

That’s all. Thank you very much for the participation!
