# Supplementary material for: A nationwide survey exploring physicians’ and pharmacists’ knowledge, awareness and perceptions regarding generic medicines in China
Source: BMC Health Serv Res. 2022 Aug 20;22:1069. doi: 10.1186/s12913-022-08438-9 (PMC9392939; doi:10.1186/s12913-022-08438-9)
Supplement: Supplementary file 3 — Additional file 3: Table S3. Perceptions regarding generic medicines between physicians and pharmacists. [file 12913_2022_8438_MOESM3_ESM.docx]

**Table S3. Perceptions regarding generic medicines between physicians and pharmacists.**

| **Characteristics** | **Mean of total perception score (SD)** | |  |
| --- | --- | --- | --- |
|  | **Physicians**  **n=1644** | **Pharmacists**  **n=4187** | ***P*-value ^*^** |
| Age(y) | | |  |
| 20-29 | 35.90 (5.74) | 37.01 (4.53) | **0.022** |
| 30-39 | 36.87 (4.85) | 36.98 (4.57) | 0.871 |
| 40-49 | 36.55 (5.15) | 36.83 (4.37) | 0.479 |
| 50-59 | 36.96 (4.98) | 37.33 (4.16) | 0.399 |
| ≥60 | 36.26 (4.83) | 37.21 (4.08) | 0.546 |
| Gender | | |  |
| Male | 37.24 (5.49) | 37.49 (4.64) | 0.868 |
| Female | 36.21 (4.66) | 36.78 (4.37) | **0.005** |
| Terminal degree | | |  |
| PhD | 36.23 (4.84) | 36.32 (4.79) | 0.637 |
| Master | 36.35 (4.95) | 36.71 (4.42) | 0.357 |
| Bachelor | 37.20 (5.17) | 37.11 (4.48) | 0.154 |
| Others | 36.10 (5.57) | 36.95 (4.33) | 0.216 |
| Professional title | | |  |
| Professor of medicine/pharmacy | 36.56 (5.04) | 37.20 (4.29) | 0.176 |
| Associate professor of medicine/pharmacy | 36.67 (5.03) | 36.76 (4.26) | 0.849 |
| Doctor/Pharmacist in charge | 36.96 (4.93) | 36.98 (4.40) | 0.733 |
| Doctor/Pharmacist | 36.38 (5.31) | 37.07 (4.63) | 0.120 |
| No title (e.g. Intern) | 36.83 (6.11) | 36.89 (4.80) | 0.615 |
| others | 36.63 (6.76) | 36.14 (4.14) | 0.907 |
| Years of experience | | |  |
| Less than 5 | 36.27 (5.60) | 36.81 (4.50) | 0.257 |
| 6-10 | 36.91 (4.91) | 37.15 (4.55) | 0.432 |
| 11-20 | 36.71 (4.92) | 36.94 (4.60) | 0.813 |
| 21-30 | 36.59 (5.34) | 36.81 (4.28) | 0.847 |
| More than 30 | 36.85 (4.63) | 37.43 (4.17) | 0.165 |
| Level of medical institution | | |  |
| Tertiary hospital | 36.46 (5.00) | 37.00 (4.46) | **0.006** |
| Secondary hospital | 36.56 (5.77) | 36.80 (4.37) | 0.771 |
| Community hospital | 37.49 (4.73) | 37.39 (4.72) | 0.773 |
| Primary health care institution | 37.66 (4.63) | 37.57 (4.80) | 0.537 |

Bold *P*-values represent statistical significance.

^*^ *P*-value calculated using Mann-Whitney U test.
